# Associations among High-Quality Protein and Energy Intake, Serum Transthyretin, Serum Amino Acids and Linear Growth of Children in Ethiopia

**DOI:** 10.3390/nu10111776

**Published:** 2018-11-16

**Authors:** Masresha Tessema, Nilupa S. Gunaratna, Inge D. Brouwer, Katherine Donato, Jessica L. Cohen, Margaret McConnell, Tefera Belachew, Demissie Belayneh, Hugo De Groote

**Affiliations:** 1 Division of Human Nutrition, Wageningen University, 6700 AA Wageningen, The Netherlands; inge.brouwer@wur.nl; 2 Ethiopian Public Health Institute, Gulele Sub City, P.O. Box 1242, Addis Ababa, Ethiopia; 3 Human Nutrition Unit, Jimma University, P.O. Box 378, Jimma, Ethiopia; teferabelachew@gmail.com; 4 Department of Nutrition Science and Public Health Graduate Program, Purdue University, West Lafayette, IN 47907, USA; gunaratna@purdue.edu; 5 Harvard T.H. Chan School of Public Health, Boston, MA 02115, USA; katherinedonato@fas.harvard.edu (K.D.); cohenj@hsph.harvard.edu (J.L.C.); mmcconne@hsph.harvard.edu (M.M.); 6 International Maize and Wheat Improvement Centre (CIMMYT), P.O. Box 5689, Addis Ababa, Ethiopia; D.Belayneh@cgiar.org; 7 International Maize and Wheat Improvement Centre (CIMMYT), P.O. Box 1041-00621, Nairobi, Kenya; h.degroote@cgiar.org

**Keywords:** protein intake, protein quality, energy intake, serum transthyretin, serum IGF-1, inflammation, linear growth, Ethiopia

## Abstract

Limited evidence is available on the associations of high-quality protein and energy intake, serum transthyretin (TTR), serum amino acids and serum insulin-like growth factor-1 (IGF-1) with linear growth of young children. Data collected during the baseline of a randomized control trial involving rural Ethiopian children aged 6–35 months (*n* = 873) were analyzed to evaluate the associations among height/length-for-age z-scores, dietary intakes, and these biomarkers (i.e., serum level of TTR, IGF-1, tryptophan and lysine, and inflammation). The prevalence of stunting was higher for children >23 months (38%) than ≤23 months (25%). The prevalence of inflammation was 35% and of intestinal parasites 48%. Three-quarters of the children were energy deficient, and stunted children had lower daily energy intake that non-stunted children (*p* < 0.05). Intakes of tryptophan, protein, and energy, and serum levels of tryptophan and IGF-1 were positively correlated with the linear growth of children. Controlling for inflammation, intestinal parasites, and sociodemographic characteristics, daily tryptophan (b = 0.01, *p* = 0.001), protein (b = 0.01, *p* = 0.01) and energy (b = 0.0003, *p* = 0.04) intakes and serum TTR (b = 2.58, *p* = 0.04) and IGF-1 (b = 0.01, *p* = 0.003) were positively associated with linear growth of children. Linear growth failure in Ethiopian children is likely associated with low quality protein intake and inadequate energy intake. Nutrition programs that emphasize improved protein quantity and quality and energy intake may enhance the linear growth of young children and need to be further investigated in longitudinal and interventional studies.

## 1. Introduction

Globally, an estimated 151 million children were affected by linear growth failure in 2017 [1]. Linear growth failure (stunting) in early childhood as a manifestation of chronic undernutrition is a major public health problem in developing countries [2]. Over 75% of all stunted children under five years of age live in either the African or Southeast Asia regions [1,2]. Linear growth failure as a result of inadequate nutrition and infections is a major cause of morbidity and mortality in infants and children [1,3]. Growth failure in early life leads to permanent impairment and can affect future generations [4]. Several studies have been conducted on the role of micronutrients in the linear growth of children [5]. However, the role of protein-energy and high-quality protein intake on linear growth of children has so far been poorly studied in developing countries [6,7]. Linear growth faltering is widespread among Ethiopian children [2,8].

**Figure 1:**
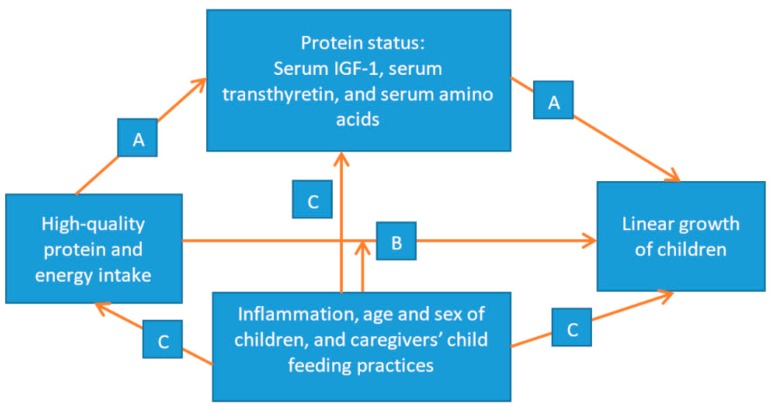
Conceptual framework depicting pathways for associations between protein and energy intakes and linear growth of children: (A) Intake of high-quality protein improves protein status [12], which in turn improves the linear growth of children; (B) The relationship between high-quality protein and energy intakes and linear growth of children is affected by inflammation [6]; and (C) Inflammation, together with characteristics of the child and caregivers’ child feeding practices, reduces nutrient (protein and energy) intake, biomarkers of protein status, and linear growth of children [12].

Protein and essential amino acids are required for the growth of children [9,10,11]. The association between children’s growth and high-quality protein intake (particularly intake of limiting essential amino acids lysine and tryptophan) is complex and influenced by several factors (see Figure 1). Childhood morbidity can cause inflammation as well as decreased appetite and can therefore reduce intake of nutrients including high-quality protein and energy. It can also lead to changes in caregivers’ child feeding practices, which can also affect nutrient intakes [12]. Children are more sensitive to high-quality protein malnutrition than adults [9,10], probably due to the high requirement for various physiological functions and additional requirements during illness. Animal-based food products contain high amounts of protein, which are considered to be of excellent quality [13,14,15]. In developing countries such as Ethiopia, however, dietary protein is mainly limited to plant-based sources, which are deficient in certain essential amino acids such as lysine and tryptophan [16,17].

The relationship between high-quality protein intake and children’s growth in the context of energy deficit as well as illness is poorly understood in developing countries [6,18]. During illness, children need additional protein and essential amino acids to recover [15,19]. The requirements for protein and essential amino acids are higher in the presence of chronic or acute infections [15,20]. Inflammation increases amino acids requirements three-fold [21]. However, the effect of inflammation on protein and amino acid requirements among children in developing countries is poorly understood [12,21]. Further, evidence suggests that energy deficit increases the need for protein and essential amino acids [22,23]. The current estimates of protein and essential amino acid requirements do not address the question of increased requirements due to frequent infections and energy deficit in children in developing countries [15].

Recent evidence suggests that stunted children might not be receiving adequate dietary intake of essential amino acids, and may have low circulating amino acids [24]. Insulin-like growth factor-I (IGF-I) is a protein hormone that mediates the effects of growth hormone and is reported to have numerous anabolic effects on skeletal muscles and other tissues [25,26,27]. When children have inadequate intake of protein and essential amino acids, their serum transthyretin (TTR), serum amino acids (AAs), as well as serum IGF-1 level maybe low, which may, in turn, reduce the growth of children. However, this relationship has not been studied in developing countries with higher levels of inflammation. Furthermore, the role of inflammation on TTR, serum essential AAs and serum IGF-1 levels among children is poorly understood in developing countries [6].

A recent study on energy supply at the country level in developing countries has shown that energy supply was correlated with stunting among children [18]. Energy deficiency caused by inadequate food intake may lead to suboptimal nutritional status. Very little information is available on the relationship between energy intake and the linear growth of children in Ethiopia. Evidence showed that the appropriate number of feedings depends on the energy density of local foods and that a higher meal frequency is needed with low energy density diets [28]. Findings on the effect of increased energy density of complementary foods on the linear growth of children have been inconsistent [29].

To our knowledge, this study is the first to investigate the associations among the intakes of protein, energy, and the essential amino acids tryptophan and lysine; serum levels of TTR, lysine, tryptophan, and IGF-1; and linear growth of Ethiopian children. The association between the growth of children and high-quality protein intake is complex and influenced by several factors (see Figure 1). Linear growth may be sensitive to intake of high-quality protein through serum transthyretin, serum amino acids, and insulin-like growth factor-1 (IGF-1) [12], and the high-quality protein requirements of children may also be affected by inflammation and low energy intake [6]. We hypothesized that high-quality protein intake, energy intake, serum TTR, serum AAs, and serum IGF-1 are associated with the linear growth of children in rural Ethiopia.

## 2. Materials and Methods

### 2.1. Study Design and Study Population

Data were collected from July–September 2015 as part of a baseline for a randomized control trial (RCT) of quality protein maize consumption of rural Ethiopian children aged 6–35 months (*n* = 873). The analysis of the main effect of the actual trial data is still ongoing. The study protocol and population characteristics have been described elsewhere [30]. A total of 1491 households were screened, of which 873 households with children aged 6–35 months were eligible and selected for data collection. Five subjects were excluded from the analysis of primary outcome (HAZ) because their records were flagged as biologically implausible anthropometric values. Of eligible children, 611 were randomly selected for biomarker sampling, and 527 stool and 537 serum samples were collected for analysis. Ethical approval was obtained from the Ethiopian Public Health Institute Scientific and Ethical Review Committee (SERO-006-02-2015) and the Harvard University Institutional Review Board (IRB14-3255). Written informed consent was obtained from all adults who were interviewed, specifically the household head and caregiver. If participants were unable to sign their name, they affixed their thumbprint to the consent form and a witness to the consent process signed the consent form.

### 2.2. Data Collection

Interviews with caregivers were conducted by trained enumerators using a pretested questionnaire which was electronically administered with tablets using Open Data Kit (University of Washingtons, Seattle, WA, USA) software. Every day, collected data were sent to the central server and transferred from comma-separated values (CSV) files into the statistical software packages.

Venous blood samples were collected from children by trained phlebotomists. About 35 g of fresh fecal samples were collected and placed in labeled clean plastic stool containers. A temporary field laboratory was set up in a central location i.e., school or health center for the laboratory technologist to immediately centrifuge and aliquot the serum into appropriate cryovials. All samples were transported for laboratory analysis promptly after collection in cold boxes containing frozen gel packs (−20 °C).

### 2.3. Dietary Assessment

Dietary recall interviews were used to estimate the amount of each food consumed by the children. High-quality protein intake was quantified as intake of lysine and tryptophan. High-quality protein intake, as well as the intakes of total protein and energy, were estimated based on the 24 h dietary recall data.

Caregivers were interviewed about the food and beverage intake of their children during the preceding 24 h defined as the time the child woke up the previous day until the time the child woke up the day of the interview. The multi-pass technique [31] was used after rigorous training and pre-tests conducted before dietary data collection. Each interview involved a stepwise series of questions, common household utensils, food substitutes (playdough, flour, lentils, and water, which were used as substitutes to estimate the quantities of the actual foods prepared and fed) and pictures of the most commonly consumed foods to improve the memory of the respondents and to assist in completing the recall. A digital food scale (Electronic Kitchen Scale EK 01) was used to measure the weight of the food consumed as well as the ingredients used in food preparation to the nearest 1 g.

First, the caregivers were asked to report everything that their children had consumed the previous day, including during the night. The opening question was; “After you got up this morning/yesterday morning, when was the first time that you had given something to eat or drink to your child?”, followed by the questions “What did your child eat or drink at that time?” and “Did the child eat or drink anything else at that time?” The same three questions were repeatedly asked until the caregiver had recalled all the food and drink items consumed over the specified period. The first pass ended with the questions “Can you remember any other times you had given something to eat or drink to your child?”. In the second pass, caregivers were asked to provide additional detailed information about each item of food and drink consumed by the children. This included the name of the food item (e.g., condiments, sugars), where they had eaten it, brand names, cooking methods, amounts served, and amount consumed. For homemade dishes, the caregivers were asked for the recipes and ingredients. The final pass reviewed all previously recalled information to confirm the accuracy of the record. During the final pass, the enumerators were also instructed to prompt for information about foods and drinks not mentioned that were considered to be easy to forget [32,33], such as snacks, fruits, water, and juices, which enumerators read from a list.

The interviews were conducted on all seven days of the week to capture variance in the intake across various days of the week. The content of protein and energy of foods consumed were obtained from the food composition databases compiled for Ethiopian National Food Consumption Survey (NFCS), which were primarily from the local food composition table (FCT) III and IV [34,35]. The values for lysine and tryptophan were borrowed from Tanzanian, UK, and the United States Department of Agriculture (USDA) food composition databases [36]. If the food was shared with other household members, FAO adult equivalent ratios were used to estimate the child’s consumption [37]. Estimated average requirement (EAR) was defined as per the World Health Organization/Food and Agriculture Organization of the United Nations (WHO/FAO) [15]. The web-based software Compl-eat© (version 1.0, Wageningen University, Wageningen, The Netherlands, http://www.compleat.nl) was used to estimate protein, energy, lysine and tryptophan intakes.

### 2.4. Anthropometrics Assessment

Anthropometrics (i.e., height or recumbent length, and weight,) were collected on all selected children. Age of children in a month was taken from their caregiver recall and further confirmed from immunization cards. The weight of children was measured with light clothing and without shoes to the nearest 100 g using a standard UNICEF SECA 874 U digital scale (UNICEF Supply Division, Copenhagen, Denmark). The scale was calibrated using standard weights after moving from one household to the next.

The length of younger children (6–23 months) was measured in a recumbent position to the nearest 0.1 cm using a measuring board designed by UNICEF (UNICEF Supply Division, Copenhagen, Denmark) with an upright wooden base and movable headpiece. The height of children older than 23 months of age was measured in a standing position with the same measuring board, to the nearest 0.1 cm.

### 2.5. Biochemical Assessment

Serum TTR, alpha-1-glycoprotein (AGP), and C-reactive protein (CRP) concentrations were determined by immune-turbidimetry using Cobas 6000 (Roche Diagnostics, GmbH, Mannheim, Germany) with fully automated clinical chemistry instruments. Inflammation was measured using CRP and AGP and defined as having either elevated CRP > 5.0 mg/L and/or AGP > 1.0 g/L. Serum IGF-1 concentrations were measured in duplicate using R&D Systems Quantikine Enzyme-linked Immunosorbent Assay (ELISA) kits (R&D Systems, Abingdon, UK) following the manufacturer’s instructions. Serum samples were pre-treated prior to analysis to dissociate or release the IGF-1 from its binding proteins. The analysis of serum amino acids (lysine and tryptophan) was conducted using Biochrom 30 amino acid analyzer (the gold standard in amino acids analysis), and the method based on ion exchange chromatography with post column derivatization with Ninhydrin, as described previously [38,39,40].

During the data collection in the field site, a portion of each stool sample was processed by the Kato-katz techniques [41], and a direct mount was prepared to diagnose the presence of active motile trophozoites and larval stages of intestinal parasites. Lugol’s iodine was added to observe cysts of the intestinal protozoan parasites. The leftover samples were preserved using 10% of formalin to preserve the morphology of the parasite ova. A portion of the preserved stool sample was analyzed with the formol-ether concentration method as described by Ritchie [42], with some modification. In brief, the stool sample was sieved with cotton gauze and transferred to a 15 mL centrifuge tube. Then 12 mL of 10% formalin and 3 mL of diethyl ether was added and centrifuged for 5 min at 1500 rpm. The supernatant was discarded and the residue was transferred to microscopic slides and observed under a light microscope at 10× and 40× magnifications for the presence of cysts and ova of the parasites. The presence of parasites was confirmed when observed by any of the methods above.

The analyses of serum transthyretin, serum IGF-1, AGP, and CRP were conducted at the Ethiopian Public Health Institute (EPHI) laboratory, certified by the Ethiopian National Accreditation Office in accordance with the requirements of ISO 17025:2005 and ISO 15189:2012. The analysis of serum amino acids was done at Ansynth Service B.V., The Netherlands, an amino acid specialized laboratory (http://www.ansynth.com/, Roosendaal, The Netherlands). The CV (inter-assay) for the various indicators were: serum transthyretin, 3.1%; IGF-1, 17%; AGP, 3.6%; CRP, 2.8%; and serum amino acids, 1.5%.

### 2.6. Statistical Analysis

Statistical analyses were conducted with SAS version 9.3 (SAS Institute, Cary, NC, USA). The weight and length of the children were converted into Z-scores for height/length-for-age (HAZ or LAZ), and weight-for-height (WHZ) according to 2006 WHO child growth standards using WHO Anthro software [43]. Stunting was defined as LAZ or HAZ scores less than 2 standard deviations below median values. The Mann–Whitney test was used to compare median high-quality protein and energy intake, serum TTR, serum IGF-1, serum lysine, and serum tryptophan between stunted and non-stunted children. Pearson correlation was used to investigate the correlation between high-quality protein intake, serum TTR, serum IGF-1, serum lysine, and serum tryptophan and linear growth of children. Multivariate linear regression was used to examine the associations between linear growth (HAZ or LAZ) as the dependent variable and serum transthyretin, serum lysine, serum tryptophan, and IGF-1 as independent variables while controlling for inflammation, intestinal parasites, age and sex of children. A *p* value <0.05 was considered statistically significant.

## 3. Results

### 3.1. Characteristics of the Study Population

The majority (96%) of caregivers were the spouse of the household head (Table 1). Caregivers’ age ranged from 22 to 34 years, with a median of 28 years. Two out of three caregivers had no formal education, and caregiver education was similar between households with stunted and those with non-stunted children. Households with stunted children were more likely to be poor (Table 1).

### 3.2. Feeding Indicators and Child Characteristics

**Figure 2:**
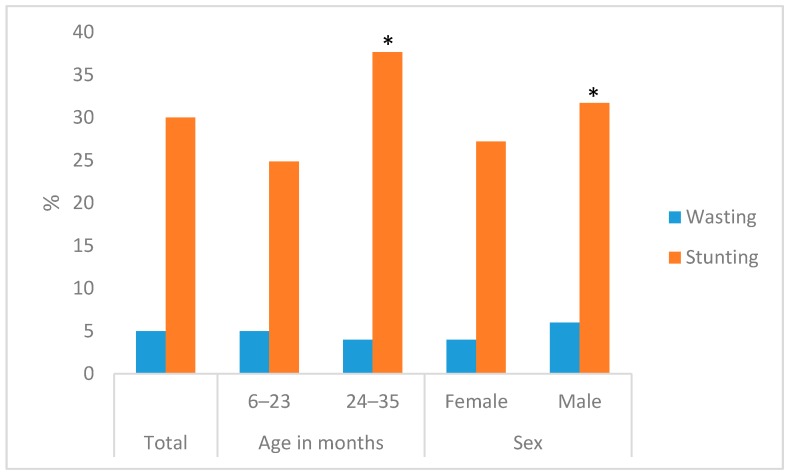
Nutritional status of children by age and sex. * *p* < 0.05, nutritional status different by age and sex.

From children who participated in the study, 48% were female and the median age was 20 months (Table 2). Most children, stunted or non-stunted, had been supplemented with vitamin A in the last six months. There were no statistical differences in reported illness among stunted and non-stunted children. About 18% of children had complaints of diarrhea; 17% of cough; and 19% of fever in the two weeks prior to the study. About 22% of children had taken drugs for intestinal worms in the six months prior to the study. There were no statistically significant differences in infant and young child feeding practices indicators between households with and without stunted children (Table S1). We found stunting was higher for older children and boys. The prevalence of wasting was about 5% (Figure 2).

### 3.3. Dietary High-Quality Protein and Energy Intake of Children

No difference was found in the median protein intake between stunted and non-stunted children (*p* > 0.05), but intake of tryptophan of stunted children was significantly lower than that of non-stunted children (Table 3).

**Figure 3:**
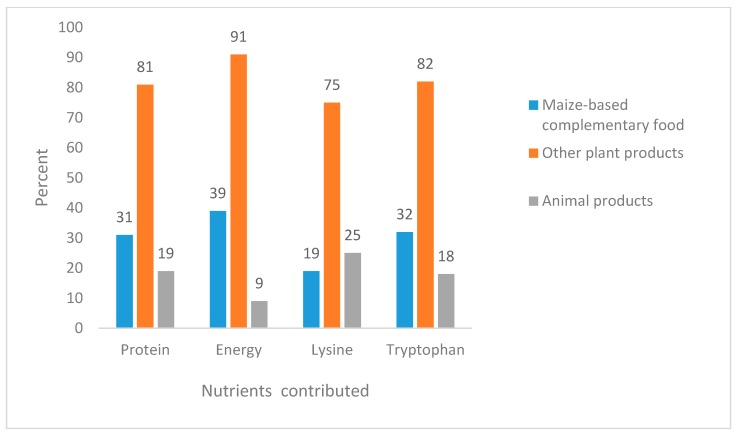
The contribution of plant- and animal-based complementary foods to nutrient intakes of children.

The energy intake of stunted children was significantly lower than that of non-stunted children. Furthermore, most children’s energy intake in both stunted and non-stunted children was below the estimated average requirement. All children with protein deficiency were also energy deficient. The median energy density of the child’s complementary foods was 1.4 kcal/g with no significant difference between stunted and non-stunted children (Table 4). We found that the contribution of cereals to the total protein and high-quality protein intake, as well as energy intake, was about 80% (Figure 3). The consumption of animal foods such as meat, poultry, and fish was very limited.

### 3.4. Protein Biomarkers of Children

Serum tryptophan and serum IGF-1 were lower for stunted than for non-stunted children (*p* < 0.005) (Table 5). No difference was found in serum lysine and serum transthyretin between stunted and non-stunted children. Over one-third of children had acute and/or chronic inflammation and about half of children had one or more intestinal parasites (Table 5).

### 3.5. Correlations among Intake of Essential Amino Acids, Serum Transthyretin, Serum Amino Acids, Serum IGF-1 and Children’s Growth

HAZ was positively correlated with intakes of tryptophan (r = 0.12, *p* < 0.0001), protein intake (r = 0.10, *p* = 0.011), energy intake (r = 0.20, *p* < 0.001), serum tryptophan (r = 0.18, *p* = 0.001) and serum IGF-1 (r = 0.12, *p* = 0.004) (Table 6). Further, we found WHZ positively correlated with serum transthyretin (r = 0.12, *p* = 0.006), and serum IGF-1 (r = 0.16, *p* = 0.0003). We found that inflammation (AGP) was negatively correlated with serum TTR (r = −0.37, *p* < 0.0001), serum tryptophan (r = −0.23, *p* < 0.0001) and serum IGF-1 (r = −0.10, *p* = 0.019).

### 3.6. Association among High-Quality Protein Intake, Energy Intake, Serum Transthyretin, Serum Amino Acids, and Serum IGF-1 with the Linear Growth (Height-for-Age, HAZ) of Children

After adjustment for inflammation status, intestinal parasites, age, sex, and household wealth, protein intake (b = 0.01, *p* = 0.005), energy intake (b = 0.0003, *p* = 0.0002), serum TTR (b = 2.58, *p* = 0.04), and serum IGF-1 (b = 0.01, *p* = 0.003) were each significantly associated with HAZ (Table 7A). While dietary intake of tryptophan per kg of body weight was positively associated with HAZ (b = 0.01, *p* = 0.001), dietary intake of lysine per kg of body weight was not (*p* = 0.69), and adjustment for inflammation status, intestinal parasites, age, sex, and household wealth resulted in no significant associations between HAZ and dietary intake or serum levels of lysine or tryptophan (*p* > 0.05, Table 7B). Dietary intake of tryptophan per kilogram body weight decreased among older children, resulting in collinearity with child age.

## 4. Discussion

We found that over one-third of children had growth failure in our study area. In Ethiopia, the prevalence of linear growth failure in children decreased from 57% in 2000 to 38% in 2015, about 1.3 percentage point reduction each year [2,8]. The existing prevalence rate still remains among the highest in the world indicating that growth failure is still a public health problem in Ethiopia, despite recent gains. In order to meet the goals for reduction in the prevalence of 40% set by the World Health Assembly [45], there is a need for country-specific evidence on the causes of child’s linear growth failure and potential interventions to address the problem. 

The present study showed that complementary foods consumed by study children were mainly prepared from cereals, suggesting low quality protein intake. The highest digestibility of protein and biological values are found in food from animal origin [18,46]. Lysine and tryptophan are considered essential amino acids because they are not synthesized by humans, and they are the most limiting essential amino acids in human diets, particularly those reliant on cereals and other plant products [16,18,24]. These two amino acids are estimated to be particularly lacking among children in sub-Saharan Africa, including Ethiopia [8], because complementary foods here are primarily maize- or otherwise plant-based [13,16,18]. Thus, interventions to improve the intake of high-quality protein from child complementary food are warranted. 

We found that three-quarters of children were energy deficient. All children with protein deficiency were also energy deficient. Furthermore, stunted children had lower daily energy intake than non-stunted children. This may suggest that children in the study area were not getting adequate food, which may have contributed to child growth failure. This is supported by a recent analysis of energy supply and children’s linear growth in developing countries, showing that total energy supply at country level was correlated with the prevalence of stunting [18]. Energy deficit in children may also lead to growth retardation, loss of fat and muscle, and increased morbidity and mortality [47]. Evidence has shown that when children experience energy restriction, there is a significant decrease in nitrogen balance and decline in IGF-1 concentrations [48]. An interventional study among Indian children found that an energy-rich low-protein supplement improved linear growth [49]. Furthermore, inadequate intake of energy in animals leads to both reduced protein synthesis and degradation of muscle protein [50]. Our data suggest that energy deficiency is a major factor limiting child growth and may result in the diversion of some protein intake to meet energy requirements. We also found that the energy density of children’s diets is reasonable as it fell in the ranges reported elsewhere for children receiving normal breastfeeding [28], indicating that the low energy intake was probably due to low food intake rather than low energy density of the food. Another possible explanation is that poor appetite is a common response to inflammation and therefore could be a major cause of low food intake by children. In view of the bulkiness of the diet, increasing the intake of complementary foods is not a feasible option in our population, and, hence, increasing energy density of consumed foods may be a better option [51]. However, there is limited recent evidence on the relationship between energy density and growth of children. A review of five studies on increased energy density of children’s complementary foods in developing countries found that only two had positive impact on the linear growth of children [29]. Further study is needed to better understand whether consumption of higher energy density food among children’s complementary foods improves linear growth.

In this study, we found that the proportion of children with total protein intake (both from diet and breast milk) below the estimated current average requirement was low (10%), while a higher proportion of children had deficient lysine intakes (30%). Protein and energy intakes were highly correlated, making it difficult to separate their relation to linear growth. Early evidence has shown that people with an energy deficit will need additional protein; even a modest energy deficit of 5% increases protein needs by about 10% [22]. The associations with protein biomarkers (serum TTR and IGF-1) suggest that there may be a biological mechanism between protein status and linear growth. Our analysis shows that over one-third of children had acute or chronic inflammation, and about half of children had one or more intestinal parasites. Although protein intake was found to be largely adequate, the current protein and essential amino acid requirements may not be adequate for energy-deficient children and for those affected by high levels of inflammation and intestinal parasites. Earlier evidence suggests that bacterial infection increases protein requirements by about 30% [15] and lysine requirements by 50% [52] in malnourished children in India. A recent study conducted among Indian school children showed that intestinal parasite infestation increased the lysine requirement by 20% [53]. Inflammation resulting from morbidities and energy deficit [20] should be taken into account when calculating the requirements for protein and essential amino acids among children in Ethiopia. Our study population is energy deficient and will, therefore, have increased protein requirements. Moreover, the children mostly consume plant-based protein with a lower utilizability. Therefore, we may conclude that our population is also protein deficient.

To our knowledge, this is the first study to assess the pattern of linear growth failure in relation to protein, lysine, tryptophan, and energy intakes, while controlling for inflammation and intestinal parasites in Ethiopia. A simple comparison between stunted and non-stunted children did not reveal a difference in the intake of protein and lysine. The regression, however, did show a significant positive association between protein intake and linear growth of children. Evidence suggests that high-quality protein has a significant impact on gene expression, especially IGF-1, which plays an important role in growth promotion [54], and in this study, serum TTR and IGF-1 were positively associated with linear growth. A recent review in developing countries showed a significant negative association between utilizable protein and stunting [18], emphasizing the need to address the low quality of dietary protein in developing countries. A longitudinal intervention study in Guatemalan children with high-protein food supplements showed an improvement in linear growth [55]. Evidence from animal trials showed that when lysine provision is inadequate, protein synthesis is unable to proceed efficiently and the rate of oxidation of all amino acids other than lysine increases disproportionately [10]. Studies in China [56] and Pakistan [57] found that fortification of wheat flour with lysine increased linear growth in children. While energy and protein intakes and biomarkers related to protein status (TTR and IGF-1) were associated with linear growth, this study did not find significant relationships between dietary or serum amino acids and linear growth. Most children in the study were breastfeeding, and breastmilk, therefore, provided a significant source of high-quality protein, particularly for younger children. Changes in dietary intake of complementary foods and breastmilk as children age may have confounded possible relationships between amino acids and linear growth. Further longitudinal study of the relationships between amino acid intakes and nutritional status are warranted, particularly as and after children cease breastfeeding.

The relationship between linear growth and serum TTR, serum lysine, and serum tryptophan were not previously studied in Ethiopia. A recent cross-sectional study among Malawian children suggests that stunted children have significantly lower circulating essential amino acids than non-stunted children [24]. In our study, we also found a positive association of the linear growth of children with serum TTR, controlled for inflammation, intestinal parasites, age and sex of children, and household wealth. Evidence has shown that serum TTR is an indicator of the availability of essential amino acids in the body [25]. Previously it was used as a tool to screen patients with high risk of protein-energy malnutrition [25]. There are several possible explanations for these positive associations. First, protein and amino acids have biological roles in protein and lipid synthesis, bone elongation, and the regulation of these and other processes necessary for linear growth [9,11,58]. Secondly, sufficient availability of amino acids potentially regulates cell and organismal growth [11]. Further, availability of amino acids is sensed via the master growth regulatory pathway of the cell, the mechanistic target of rapamycin complex 1 (mTORC1) [11,59], that will stimulate protein synthesis, cell, and organismal growth when amino acids are sufficient [60]. Inadequate dietary intakes of protein and essential amino acids may adversely affect serum amino acid status, which may, in turn, reduce the growth of children. Our data, however, did not show an association between serum lysine and tryptophan and linear growth of children. This needs further investigation.

The association between serum IGF-1 concentration and linear growth of children in developing countries is poorly understood. IGF-1 is a growth-promoting polypeptide that is essential for normal growth and development of children [27]. It is a major regulator of muscle protein and glucose homeostasis [26]. IGF-1 is also an important growth hormone, mediating protein anabolism and linear growth [27]. IGF-1 serum levels are responsive to improved nutritional status [61] and high-protein intake [48,62]. Protein restriction in children results in declined IGF-I concentrations [48]. Evidence from an animal model suggests that loss of IGF-1 signaling impairs muscle growth [26] and inactivation of IGF-1 causes linear and radial skeletal growth retardation [63]. We also found that serum IGF-1 concentration was positively associated with the linear growth of children. Possible reasons are that low quantity and quality protein intake might affect stimulation of serum IGF-1, which mediates the linear growth of children. However, further longitudinal interventions studies are needed to understand the stimulating effect of high-quality protein intake on serum IGF-1 and children’s linear growth.

This study has several limitations. First, we could not establish causality between the observed associations, because of the cross-sectional character of the study. Second, the present study did not measure all factors that may be important for children’s linear growth, e.g., environmental factors related to health and hygiene or child caregiving practices and resources [64]. Third, the misreporting of food consumption is a potential issue for all dietary assessment methods, and it is not known to what extent parents underreport the dietary intakes of young children. The potential for underestimating leftovers, resulting in over-reporting of actual consumption, is a particular risk in this age group [65].

## 5. Conclusions

Inadequate protein and energy intake may be a predictor of childhood linear growth failure in rural Ethiopia. Nutrition programs that emphasize food security, recommended child feeding practices, and increased nutrient density of complementary foods, including density of high-quality protein and energy, may improve child’s linear growth, especially in areas characterized by high inflammation and infections. Further, the calculated requirements for protein and essential amino acid intakes for children should account for inflammation, energy deficiency, and intestinal parasites in Ethiopia. The effect of consumption of high-quality protein food on linear growth in children will have to be further investigated in longitudinal intervention studies, including whether consumption of high-quality protein enriched complementary foods, such as cereals with increased protein quality (e.g., quality protein maize), increase serum transthyretin and serum amino acid status, which in turn may lead to improved linear growth.

## Figures and Tables

**Table 1:** Socioeconomic and demographic characteristics of participating households.

Indicators	All Households (*n* = 868)	Households with Stunted Child (*n* = 258)	Households with Non-Stunted Child (*n* = 610)
Caregiver age (years), Median (Q1, Q3)	28 (25, 32)	28 (25, 32)	28 (25, 32)
Caregiver relationship to the household head, %			
Household head	3	2	4
Spouse	96	97	96
Other	1	1	-
Caregiver with no formal education, %	65	67	64
Religion, %			
Christian	62	61	63
Muslim	38	39	37
Family size, Median [Q1, Q3]	6 [5, 8]	6 [5, 7]	6 [5, 8]
Wealth tertiles ^1^, %			
1st tertile (poorer)	33	37	31 *
2nd tertile	33	37	32 *
3rd tertile (wealthier)	33	26	37 *

* *p* < 0.05, households with stunted different from households without stunted children. ^1^ Wealth tertiles were constructed based on household assets using principal component analysis (PCA) techniques and the list variables used for wealth tertiles were sickle, hoe, shovel, axe, knap sack spray, ox plough, horse or mule cart, donkey or oxen cart, horse or mule saddle, bicycle, motor bike, car track, grinding stone, motorized, charcoal, kerosene, water carrier, refrigerator, watch clock, table, chair, bed, electric,, kerosene, radio, tape player, mobile phone, non-mobile phone, television, and owned land.

**Table 2:** Children’s health characteristics.

Variables	Total (*n* = 868)	Stunted (*n* = 258)	Non-Stunted (*n* = 610)
Female, %,	48	44	49
Age in months, Median (Q1, Q3)	20 (13, 27)	23 (16, 28)	19 (12, 26)
Vitamin A supplementation in the last six months, %	83	84	83
Any multivitamin in the last six months, %	4	6	4
Iron tablets/syrups in the last six months, %	1	2	1
Any drugs for intestinal worms in the last six months, %	22	24	21
Diarrhea in the two weeks before survey, %	18	17	18
Cough or breathing problems in the two weeks before the survey, %	17	15	18
Fever in the two weeks before the survey, %	19	20	19
HAZ (overall), Mean ± SD	−1.3 ± 1.3	−2.8 ± 0.7	−0.7 ± 1.0

**Table 3:** Dietary protein and essential amino acids intake of children ^1^.

Variables	Total (*n* = 868)	Stunted (*n* = 258)	Non-Stunted (*n* = 610)
Protein intake (g/day) ^2^	16 (12, 22)	16 (11, 21)	16 (12, 22)
Lysine intake (mg/day) ^2^	589 (349, 859)	541 (333, 813)	597 (356, 868)
Tryptophan intake (mg/day) ^2^	233 (164, 343)	205 (142, 284)	246 (173, 369) *
Proportion of children with low protein intake (below EAR), % ^3^	10.5	10	11
Proportion of children with low lysine intake (below EAR), % ^3^	31	30	31
Proportion of children with low tryptophan intake (below EAR), % ^3^	4	4	4

* *p* < 0.001, stunted different from non-stunted children, tested with Mann–Whitney test. ^1^ Intake includes both diet and breast milk. ^2^ Median [25th, 75th]. ^3^ The recommended EARs [44] are: protein (0.87 g/(kg·d)); Lysine (45 mg/(kg·d)); Tryptophan (6 mg/(kg·d)); energy (678 kcal, 764 kcal and 935 kcal for children aged 6–8 months, 9–11 months and 12–23 months, respectively).

**Table 4:** Energy intake of children ^1^.

Variables	Total (*n* = 868)	Stunted (*n* = 258)	Non-Stunted (*n* = 610)
Energy intake (kcal/day) ^2^	695 (519, 870)	643 (463, 818)	703 (550, 891) *
Proportion of children with low energy intake (below EAR), % ^3^	76	85	72 *
Energy density (kcal/g) ^2^	1.4 (1.2, 1.6)	1.4 (1.2, 1.6)	1.3 (1.2, 1.6)

* *p* < 0.001, stunted different from non-stunted children, tested with Mann–Whitney test. ^1^ Intake includes both diet and breast milk. ^2^ Median [25th, 75th]. ^3^ The recommended EARs [44] are: (678 kcal, 764 kcal and 935 kcal for children aged 6–8 months, 9–11 months and 12–23 months, respectively).

**Table 5:** Protein and inflammation biomarkers and intestinal parasites of children ^1^.

Variables ^1^	Total (*n* = 868)	Stunted (*n* = 258)	Non-Stunted (*n* = 610)
Serum transthyretin (g/L)	0.17 (0.14, 0.20)	0.17 (0.14, 0.19)	0.17 (0.14, 0.21)
Serum IGF-1 (ng/mL)	30 (22, 44)	26 (19, 36)	32 (23, 46) *
Serum lysine (µmol/L)	141 (116, 164)	138 (116, 159)	142 (117, 167)
Serum tryptophan (µmol/L)	42 (32, 51)	39 (23, 49)	42 (34, 51) *
AGP (g/L)	0.84 (0.65, 1.12)	0.83 (0.65, 1.11)	0.85 (0.65, 1.12)
CRP (mg/L)	0.67 (0.32, 2.03)	0.75 (0.32, 1.77)	0.65 (0.31, 2.11)
Prevalence of inflammation (acute and/or chronic), % ^2^	35	35	35
Prevalence of one or more intestinal parasites, %	48	50	46

* *p* < 0.05, stunted different from non-stunted children checked by Mann–Whitney test. CRP: C-reactive protein. AGP: α-1-glycoprotein protein concentration. ^1^ Values are Median [25th, 75th] unless stated otherwise. ^2^ Inflammation: CRP > 5 mg/L and/ or AGP > 1 g/L.

**Table 6:** Pearson correlations between child’s growth and other variables.

Indicators	HAZ	WHZ	Serum Transthyretin (g/L)	Serum Lysine (µmol/L)	Serum Tryptophan (µmol/L)	Serum IGF-1 (ng/mL)	AGP (g/L)	CRP (mg/L)	Lysine Intake (mg/kg/Day)	Tryptophan Intake (mg/kg/Day)	Protein Intake (g/Day)	Energy Intake (kcal/Day)	Intestinal Parasites
WHZ	0.11 **												
Serum transthyretin (g/L)	0.08	0.12 **											
Serum lysine (µmol/L)	0.06	0.04	0.13 *										
Serum tryptophan (µmol/L)	0.18 **	0.01	0.25 ***	0.55 ***									
Serum IGF-1 (ng/mL)	0.12 **	0.16 **	0.22 ***	0.02	0.07								
AGP (g/L)	−0.02	−0.02	−0.37 ***	−0.11 *	−0.23 ***	−0.1 ***							
CRP (mg/L)	0.005	−0.05	−0.32 ***	−0.13 *	−0.11 ***	−0.08 *	0.51 ***						
Lysine intake (mg/kg/day)	−0.01	−0.1 *	−0.03	0.01	−0.08	−0.09	0.01	0.09					
Tryptophan intake (mg/kg/day)	0.12 **	−0.069	−0.055	0.071	0.074	−0.042	−0.002	0.019	0.68 ***				
Protein intake (g/day)	0.10 *	0.06	−0.01	0.04	−0.05	−0.07	0.03	0.04	0.74 **	0.47 **			
Energy intake (kcal/day)	0.13 **	0.07	−0.02	0.01	−0.03	−0.08	0.01	0	0.68 **	0.55 **	0.88 ***		
Intestinal parasites	−0.05	−0.1 ***	−0.05	0.05	−0.01	0.01	0.03	0.04	0.01	0.03	−0.03	−0.01	
Wealth index	0.1 **	0.11 **	0.01	0.04	0.01	0.04	0.004	0.05	0.09 *	0.03	0.01	0.01	0.01

***: *p* < 0.001, **: *p* < 0.01, * *p* < 0.05. HAZ: Height-for-age Z-score. WHZ: Weight-for-height-Z-score. CRP: C-reactive protein. AGP: α-1-glycoprotein protein concentration. IGF-1: insulin-like growth factor-1.

**Table 7:** (**A**) The relationships of protein and energy intake, serum transthyretin, and serum IGF-1 with the linear growth (height-for-age, HAZ) of children; (**B**) The relationships of lysine and tryptophan intake and serum lysine and tryptophan with linear growth (height-for-age, HAZ) of children.

**(A)**																		
**Models**	**Model 1**			**Model 2**			**Model 3**			**Model 4**								
**Fixed Effects**	**b**	**SE**	***p***	**b**	**SE**	***p***	**b**	**SE**	***p***	**b**	**SE**	***p***						
Intercept	−1.04	0.24	<0.0001	−1.10	0.26	<0.0001	−1.07	0.33	0.001	−0.89	0.24	0.0002						
Protein intake (g/day)	0.01	0.005	0.01															
Energy intake (kcal/day)				0.0003	0.0002	0.04												
Serum transthyretin (g/L)							2.58	1.24	0.04									
Serum IGF-1 (ng/mL)										0.01	0.003	0.0004						
Serum AGP(g/L)	0.11	0.18	0.54	0.11	0.18	0.53	−0.06	0.16	0.70	−0.12	0.16	0.44						
Serum CRP (mg/L)	−0.001	0.01	0.88	−0.0005	0.01	0.92	0.01	0.005	0.25	0.004	0.005	0.37						
Intestinal parasites	−0.07	0.12	0.55	−0.08	0.12	0.51	−0.10	0.12	0.40	−0.08	0.11	0.47						
**(B)**																		
**Models**	**Model 1**			**Model 2**			Model 3 *			Model 4 *			Model 5 *			Model 6 *		
**Fixed effects**	**b**	**SE**	***p***	**b**	**SE**	***p***	**b**	**SE**	***p***	**b**	**SE**	***p***	**b**	**SE**	***p***	**b**	**SE**	***p***
Intercept	−1.26	0.09	<0.0001	−1.549	0.0948	<0.0001	−0.70	0.24	0.004	−0.63	0.29	0.03	−0.40	0.39	0.31	−0.92	0.39	0.02
Lysine intake per kg body weight (mg/kg/day)	−0.0004	0.001	0.69				−0.002	0.001	0.11									
Tryptophan intake per kg body weight (mg/kg/day)				0.01	0.003	0.001				−0.004	0.003	0.23						
Serum lysine (µmol/L)													−0.0003	0.002	0.89			
Serum tryptophan (µmol/L)																0.01	0.005	0.10
Serum AGP(g/L)							0.10	0.18	0.58	0.10	0.18	0.58	−0.32	0.19	0.09	−0.25	0.19	0.20
Serum CRP (mg/L)							0.0002	0.01	0.96	−0.00002	0.01	1.00	0.01	0.01	0.13	0.01	0.01	0.14
Intestinal parasites							−0.08	0.12	0.52	−0.07	0.12	0.57	−0.01	0.14	0.96	−0.01	0.14	0.95

(**A**): All models were adjusted for sex of child, age of child (in months), and household wealth tertile. (**B**) * Model was adjusted for sex of child, age of child (in months), and household wealth tertile.

## Supplementary Materials

The following are available online at http://www.mdpi.com/2072-6643/10/11/1776/s1, Table S1: Infant and young child feeding indicators.
