# Supplementary material for: Associations among High-Quality Protein and Energy Intake, Serum Transthyretin, Serum Amino Acids and Linear Growth of Children in Ethiopia
Source: Nutrients. 2018 Nov 16;10(11):1776. doi: 10.3390/nu10111776 (PMC6266228; doi:10.3390/nu10111776)
Supplement: Supplementary file 1 [file nutrients-10-01776-s001.pdf]

Table S1: Infant and Young child Feeding Indicators

Variables	Total (n=868)	Stunted (n=258)	Non-stunted (n=610)
Prevalence of child ever being breastfed, %	99.7	99.6	99.7
Prevalence of initiation of breastfeeding (within 1 hour), %	71	67	73
Prevalence of colostrum/ first milk feeding, %	74	71	76
Prevalence of pre-lacteal feeding, %	3	4	3
Prevalence of exclusively breastfeeding for 6 months, %	53	53	53
Complementary feeding started At 6 month, %	50	50	49
Prevalence of bottle feeding, %	4	4	4
